# Challenges and concepts in the diagnosis and management of ocular graft-versus-host disease

**DOI:** 10.3389/fmed.2023.1133381

**Published:** 2023-02-16

**Authors:** Christoph Tappeiner, Arnd Heiligenhaus, Joerg P. Halter, Elisabetta Miserocchi, Francesco Bandello, David Goldblum

**Affiliations:** ^1^Department of Ophthalmology, Pallas Klinik, Olten, Switzerland; ^2^Department of Ophthalmology, University Hospital Essen, University Duisburg-Essen, Essen, Germany; ^3^Department of Ophthalmology, San Raffaele Scientific Institute, University Vita-Salute, Milan, Italy; ^4^Faculty of Medicine, University of Bern, Bern, Switzerland; ^5^Department of Ophthalmology at St. Franziskus Hospital, Muenster, Germany; ^6^Department of Hematology, University Hospital Basel, University of Basel, Basel, Switzerland; ^7^Faculty of Medicine, University of Basel, Basel, Switzerland

**Keywords:** ocular graft-versus-host disease (GVHD), dry eye, ocular surface inflammation, diagnosis, treatment

## Abstract

Graft-versus-host disease (GVHD) is characterized by tissue inflammation in the host following an allogeneic hematopoietic cell transplantation (HCT). The pathophysiology is complex and only incompletely understood yet. Donor lymphocyte interaction with the histocompatibility antigens of the host plays a crucial role in the pathogenesis of the disease. Inflammation may affect multiple organs and tissues, e.g., the gastrointestinal tract, liver, lung, fasciae, vaginal mucosa, and the eye. Subsequently, alloreactive donor-derived T and B lymphocytes may lead to severe inflammation of the ocular surface (i.e., cornea and conjunctiva) and the eyelids. Furthermore, fibrosis of the lacrimal gland may lead to severe dry eye. This review focuses on ocular GVHD (oGVHD) and provides an overview of current challenges and concepts in the diagnosis and management of oGVHD. Ophthalmic manifestations, diagnostic procedures, grading of severity and recommendations for ophthalmic examination intervals are provided. Management of ocular surface disease with lubricants, autologous serum eye drops, topical anti-inflammatory agents and systemic treatment options are described based on the current evidence. Ocular surface scarring and corneal perforation are severe complications of oGVHD. Therefore, ophthalmic screening and interdisciplinary treatment approaches are highly relevant to improve the quality of life of patients and to prevent potentially irreversible visual loss.

## 1. Introduction

Graft-versus-host disease (GVHD) is a severe complication after allogeneic hematopoietic cell transplantation (HCT). Tissue inflammation in the host due to donor lymphocyte interaction with the histocompatibility antigens of the host may lead to a high morbidity and even mortality in these patients. This review focuses on ocular GVHD (oGVHD) and provides an overview of current challenges and concepts in the diagnosis and management of oGVHD.

### 1.1. Definition of GVHD

Allogeneic HCT offers the best chance of cure for several malignant hematological as well as non-malignant disorders like bone marrow failure, hemoglobinopathies or immunodeficiencies. Currently. over 30,000 allogeneic HCT are performed annually worldwide with over 18,000 in Europe in 2020 (1).

GVHD is one of the most important causes for non-relapse mortality post-transplantation. The current understanding of the pathophysiologic concepts and therapeutic targets has tremendously expanded during the last 20 years and recently been summarized in three excellent reviews (2–4). Chronic GVHD is the most common long-term complication after allogeneic HCT with an important impact on survival, morbidity, and quality of life. Traditionally, acute and chronic GVHD was differentiated depending on the time of the initial manifestation before or after 100 days post-transplant. These criteria were revised in the 2005 and 2014 National Institute of Health (NIH) Consensus Conference, introducing new criteria/definition for acute and chronic GVHD (5–7). Acute GVHD is defined as an immediate multi-organ inflammatory syndrome following HCT primarily affecting the skin, liver, and digestive tract, whereas chronic GVHD is a pleiotropic, multi-organ syndrome characterized by tissue inflammation and fibrosis that involves multiple sites including the skin, lungs, liver, gastrointestinal tract, mouth, genitalia, and eyes (5–8). Accordingly, the diagnosis of chronic GVHD requires at least one diagnostic sign of chronic GVHD or a distinctive manifestation plus a pertinent biopsy or another test (e.g., Schirmer test, evaluation by an ophthalmologist) showing or confirming chronic GVHD (Table 1).

**Table 1:** Criteria for clinical trials in chronic graft-versus-host disease.

1. Distinction from acute GVHD.
2. Presence of at least one diagnostic clinical sign of chronic GVHD or presence of at least one distinctive manifestation confirmed by pertinent biopsy or other relevant tests.
- Diagnostic signs:
- Skin: poikiloderma, lichen planus-like eruptions, deep sclerosis, morphea-like superficial sclerotic features, lichen sclerosus-like lesions
- Mouth: lichen planus-like changes
- Genitalia: lichen planus-like features, lichen sclerosus-like features, females: vaginal scarring or clitoral/labial agglutination, males: phimosis or urethral/meatus scarring or stenosis
- Gastrointestinal tract: esophageal web, strictures or stenosis in upper or mid third of esophagus
- Lung: bronchiolitis obliterans by biopsy
- Muscle and fascia: fasciitis, joint stiffness, or contractures from sclerosis
- Distinctive signs:
- Skin: depigmentation, papulosquamous lesions
- Nails: dystrophy, longitudinal ridging, splitting or brittle features, onycholysis, pterygium unguis, nail loss
- Scalp and body hair: new onset scarring or nonscarring scalp alopecia, scaling, loss of body hair
- Mouth: xerostomia, mucocele, mucosal atrophy, pseudomembranes, ulcers
- Eyes: new onset gritty or painful eyes, cicatricial conjunctivitis, keratoconjunctivitis sicca, confluent areas of punctate keratopathy
- Genitalia: erosions, fissures, ulcers
- Lung: air trapping and bronchiectasis on chest CT
- Muscle and fascia: myositis or polymyositis
3. Exclusion of other possible diagnoses.
Diagnosis of chronic graft-versus-host disease according to the NIH consensus development project (7). Scoring of organ manifestations requires careful assessment of signs, symptoms, laboratory values, and other study results. A clinical scoring system (0–3) is provided for evaluation of the involvement of individual organs and sites. The proposed global assessment of severity (mild, moderate, or severe) is derived by combining organ and site-specific scores.

### 1.2. Epidemiology of GVHD

After the first HCT in 1968 survival rates have increased in the last decades, due to human leukocyte antigen (HLA) matching, continuously improved preconditioning protocols and immunosuppressive regimen (7, 9, 10). Both, acute and chronic GVHD occur in about 30%–70% of patients after HCT depending on transplant regimens and GVHD prophylaxis strategies (7, 11). A variety of risk factors for GVHD related to donor as well as to recipients’ characteristics have been identified. The most important are the degree of histocompatibility, the source of hematopoietic progenitor cells, sex mismatch (transplantation from female donor to male recipient), the intensity of conditioning and immunosuppression, the age of donor and recipient and for chronic GVHD prior acute GVHD (2, 3, 8, 12–14).

### 1.3. Definition of ocular GVHD

Different criteria for the diagnosis of oGVHD have been proposed in the last decades (8). The original NIH criteria defined new onset of dry eye after HCT documented by low Schirmer test values with a mean value of both eyes <5 mm at 5 min or a new onset of keratoconjunctivitis sicca by slit-lamp examination with mean values of 6 to 10 mm at 5 min on the Schirmer test as sufficient for the diagnosis of chronic oGVHD if accompanied by distinctive manifestations in at least one other organ (6). An international consensus group proposed criteria based on Ocular Surface Disease Index (OSDI), Schirmer test score without anesthesia, corneal fluorescein staining and conjunctival injection (15). A score of 4–5 and ≥ 6 indicates probable or definite oGVHD, accordingly (15).

### 1.4. Epidemiology of ocular GVHD

Acute GVHD has been reported in 40%–50% of HCT patients (16). Ocular affection in acute GVHD is quite rare and has been reported in about 7.2% after HCT (17, 18). On the other hand, occurrence of chronic oGVHD was observed in 30%–60% in the further course after HCT (19, 20), and in 60%–90% of patients with systemic GVHD (7, 10, 19, 21, 22). Lower incidences have been found in Asian studies (23–25). The mean latency of oGVHD after HCT is about 1.5 years (26). Cumulative increase of incidences over time after HCT has been reported, with a prevalence of 16% by 100 days and 35% after 2 years (21). In children, symptoms consistent with chronic oGVHD have been found at highly variating rates from 4% up to 62% (27–33). In a large prospective study, a total of 29.4% of patients with chronic oGVHD were identified using the NIH consensus criteria (34).

## 2. Pathophysiology of GVHD and ocular GVHD

**Figure 1:**
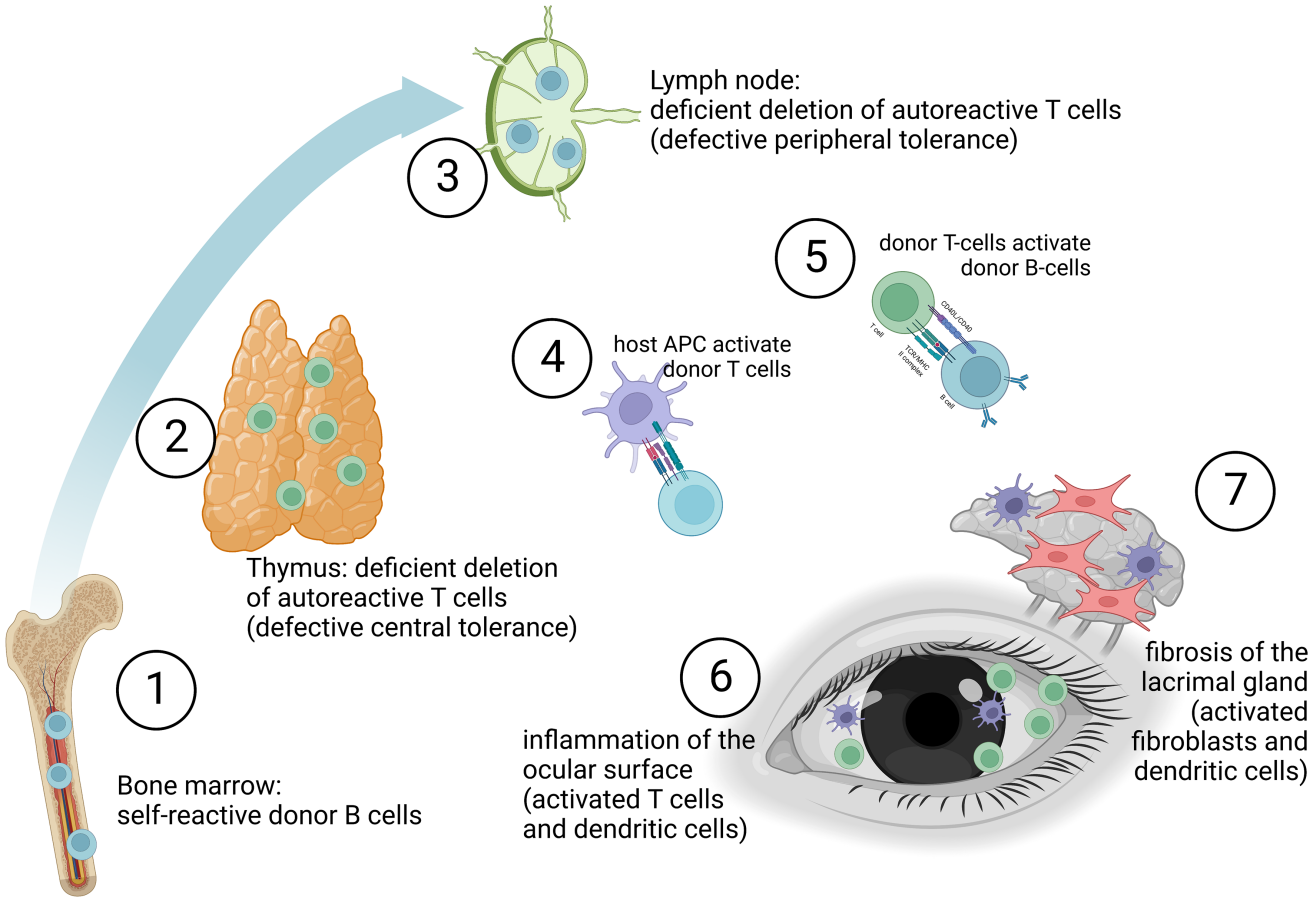
Graft-versus-host disease may be due to self-reactive donor B cells (1), deficient deletion of autoreactive donor T cells in the thymus (2) or deficient deletion of autoreactive donor T cells in the lymph nodes (3). Especially antigen-presenting cell (APC) driven activation of donor T cells (4) but also B cells (5) lead to an inflammation of the ocular surface (6). Furthermore, activation of fibroblasts by APCs (e.g., dendritic cells) induces fibrosis of the lacrimal gland (7). (The figure was created with biorender.com.)

Pre-clinical animal models have been critical not only in understanding the immune mechanisms of systemic but also oGHVD (35–37). Acute and chronic GVHD are immune-mediated diseases involving a variety of immune cells such as macrophages, T cells and B cells (11, 19, 38, 39). Figure 1 depicts the immunological activation leading to ocular surface inflammation and lacrimal gland fibrosis. Self-reactive T cells (CD4+ and CD8+), deriving from the donor, are insufficiently deleted in the thymus (defective central tolerance) and in the lymph nodes (defective peripheral tolerance). These T cell mediated immune response is directed against host antigens as major (MHC) and minor (miHAG) histocompatibility antigens (40). The response is driven mainly by differences in host and donor antigen expression, e.g., by HLA mismatch (41, 42). But even in HLA-matched HCT, differences in polymorphic minor histocompatibility antigens (miHAs) and specific miHAs may trigger GVHD (43, 44). Imbalance between effector and regulatory T cells functions triggers the inflammatory cascades (11, 45–47). Although also B cells and antigen-presenting cells (APC) are involved, donor T cells are probably the predominant factor in the orchestration of systemic and ocular disease (48). In oGVHD, activation of APC, differentiation, proliferation and activation of donor T cells, and activation of B cells with release of pro-inflammatory cytokines currently are supposed to induce and maintain inflammation in the ocular surface, to activate fibroblasts and dendritic cells in the lacrimal gland finally leading to lacrimal tissue fibrosis (49, 50). However, tissue damage in oGHVD is not limited to the ocular surface and the lacrimal gland. Recent pre-clinical and clinical studies have shown that ocular adnexa are involved and Meibomian gland and ocular surface damage correlate with each other (51).

## 3. Risk factors for the occurrence of ocular GVHD

A variety of risk factors associated with the onset of oGVHD have been reported (52), e.g., previous acute GVHD (21, 25), use of peripheral blood stem cells (25, 53), transplantation from a female donor to a male recipient (21, 54), absence of anti-thymocyte globulin prophylaxis (25), larger number of organs and tissues involved with GVHD (25, 55), and non-Caucasian and EBV-seropositive donors (56). Other risk factors are mismatch of HLA antigens, higher donor or recipient ages, and diabetes mellitus (25, 57). Increased occurrence of oGVHD has been found in patients with involvement of the skin (20, 21, 58), oral mucosa (20, 58), liver (56), or gastrointestinal tract and pulmonal involvement in chronic GVHD (25). Furthermore, ethnicity may have an impact, with Caucasians being at lower risk than Asians (56). Cord blood cell transplants (53), *in vitro* or *in vivo* T cell depletion or posttransplant cyclophosphamide lower the risk for GVHD. Dry eye and Meibomian gland disease before HCT may also be a risk factor for oGVHD, or worsen after GVHD (59–62).

## 4. Grading of ocular GVHD

Several grading systems have been proposed for oGVHD, which are based to varying degrees on findings by ophthalmologists or patient-reported symptoms. The international chronic oGVHD Consensus group (ICCGVHD) introduced criteria for the diagnosis of chronic oGVHD, based on scores calculated by ocular surface disease index (OSDI), Schirmer test without anesthesia, corneal fluorescein staining, conjunctival injection and the presence or absence of systemic GVHD (15, 63). On the other hand, the NIH chronic GVHD consensus group eye score system classifies oGVHD according to the degree of symptoms of dry eye (grade 1: mild dry eye symptoms not affecting activities of daily living (ADL) OR asymptomatic signs of keratoconjunctivitis sicca; grade 2: moderate dry eye symptoms partially affecting ADL (requiring drops >3x per day or punctal plugs), without vision impairment; grade 3: severe dry eye symptoms significantly affecting ADL (special eyewear to relieve pain) or unable to work because of ocular symptoms or loss of vision caused by keratoconjunctivitis sicca) (6, 7). A subsequent study aimed for validation of the suggested measurement scales. Herein, clinician or patient-reported changes in eye symptoms with calculated changes in 5 candidate scales (NIH eye score, patients-reported global rating of eye symptoms, Lee eye subscale, Ocular Surface Disease Index (OSDI), and Schirmer test) were compared. The results supported the use of the NIH eye score as a sensitive measures of eye symptom changes in clinical trials assessing treatment of chronic GVHD (64). Subsequently, the NIH chronic GVHD diagnosis and staging system criteria were refined with emphasis placed on usage of lubricant eye drops for dryness symptoms (65). Further scoring systems have been proposed by Robinson et al. based on exemplary photographs for everted upper and lower eyelids showing the different grades of conjunctival inflammation associated with chronic oGVHD (66). Furthermore, the ICCGVHD has proposed a grading system for conjunctival involvement (15, 67).

## 5. Recommendations for screening

Importantly, risk factors for ocular involvement have been investigated. In children, multiorgan GVHD involvement including skin and lung disease, and patients with ocular discomfort are at increased risk for eye involvement (27). However, as a significant number of GVHD patients do not exploit overt symptoms of eye involvement, regular ophthalmic screenings are recommended.

For early diagnosis of oGVHD, comprehensive ophthalmic evaluations by ophthalmologists are generally recommended before and after allogeneic HCT (68). In the acute phase, intervals corresponding to disease severity are recommended.

Chronic oGVHD may significantly influence quality of life (22). However, symptoms of chronic oGVHD may be subtle. Onset of any eye symptoms should prompt ophthalmic evaluation. More severe ocular surface damage at baseline indicates an increased risk to subsequent worsening and impaired vision (69). Therefore, prevalent ocular surface alterations and dry eye states should be evaluated in advance. Taken previous considerations, screening should be instituted at 3 (at the latest 6) months following transplantation (70, 71), and annually afterwards. Importantly, the screening intervals should be adapted to disease severity. There are no specific symptoms of oGHVD that allow a reliable differentiation from “simple” dry eye disease or lacrimal gland damage by total body irradiation. Therefore, any worsening or new manifestation of dry eye symptoms and/or worsening or new onset of ocular surface disease in patients after HCT should be evaluated and monitored closely.

Simple self-testing may further be critical for screening. For ocular discomfort testing, the ocular surface disease index (OSDI) questionnaire – considering vision-related function, ocular symptoms, and environmental triggers—may be used (72), and daily lubricant use reported. According to a recent study, the OSDI questionnaire is a valid screening test for oGVHD in transplant clinics and for patients’ self-monitoring (73). Thus, screening intervals may be adjusted based on the results from the OSDI questionnaire. The OSDI and other questionnaires are described in more detail in section 6.3.

## 6. Diagnosis of ocular GVHD

### 6.1. Ocular symptoms and findings

In the absence of overt ocular symptoms and signs during the acute disease stage, diagnosis may be delayed. Disease may partially mimic other immune-mediated inflammatory processes of the ocular surface. While no pathognomonic symptoms or clinical signs of oGVHD have been defined, certain combinations of findings are frequently present, and are provided within several recent publications (15, 67; Table 2). Key features of disease are new onset of refractory dry eye, being the most frequent manifestation (40%–70%), and secondary ocular surface damage (52). Patients suffer from diverse symptoms of the autoinflammatory reaction (particularly dry eye), including irritation, pain, burning, dryness, itchiness, blurred vision, foreign body sensation, photophobia, and redness (70, 74, 75). Visual disturbance may be the consequence from corneal higher order aberrations resulting from corneal pathology (76).

**Table 2:** Ophthalmological findings in ocular GVHD patients.

Localization	Findings
General	Pseudoptosis, frequent blinking, photophobia, decreased vision
Lacrimal glands	Dry eye disease
Lacrimal duct	Punctal occlusion
Lids	Periorbital hyperpigmentation, Meibomian gland dysfunction, anterior/posterior blepharitis, telangiectasias, entropion, dis/trichiasis, keratinization
Conjunctiva	Hyperemia, exudation (serous, hematogenous), chemosis, fibrosis, pseudomembranes, scarification, lid-parallel conjunctival folds (LIPCOF)
Cornea	Punctate keratopathy, filaments, erosion, vascularization, scarring, thinning, ulceration, perforation, calcification
Sclera	Episcleritis, scleritis
Intraocular	Cataract, uveitis (retinitis), retinal hemorrhage, papilledema

Severe ocular discomfort from dry eye, corneal epitheliopathy by means of fluorescein staining and vision loss are resulting in impaired quality of life (22). Patients with oGVHD had worse quality of life than patients without ocular involvement (77). In clinical studies, symptoms are quantified using validated QOL instruments such as Ocular Surface Disease Index (OSDI), National Eye Institute Visual Function Questionnaire (NEI-VFQ-25), and Symptom Assessment in Dry Eye (SANDE). Respective studies show that disease impact on QOL was comparable to herpetic uveitis or retinal vein occlusion (22).

By en-face evaluation, photophobia, pseudoptosis, frequent blinking or periorbital hyperpigmentation may be seen. Findings at the lid margin are common in oGVHD. Blepharitis and Meibomian gland dysfunction (50%) are probably the first signs of disease. Subsequently, atrophy, irregularity and keratinization of the eyelid margin may occur.

**Figure 2:**
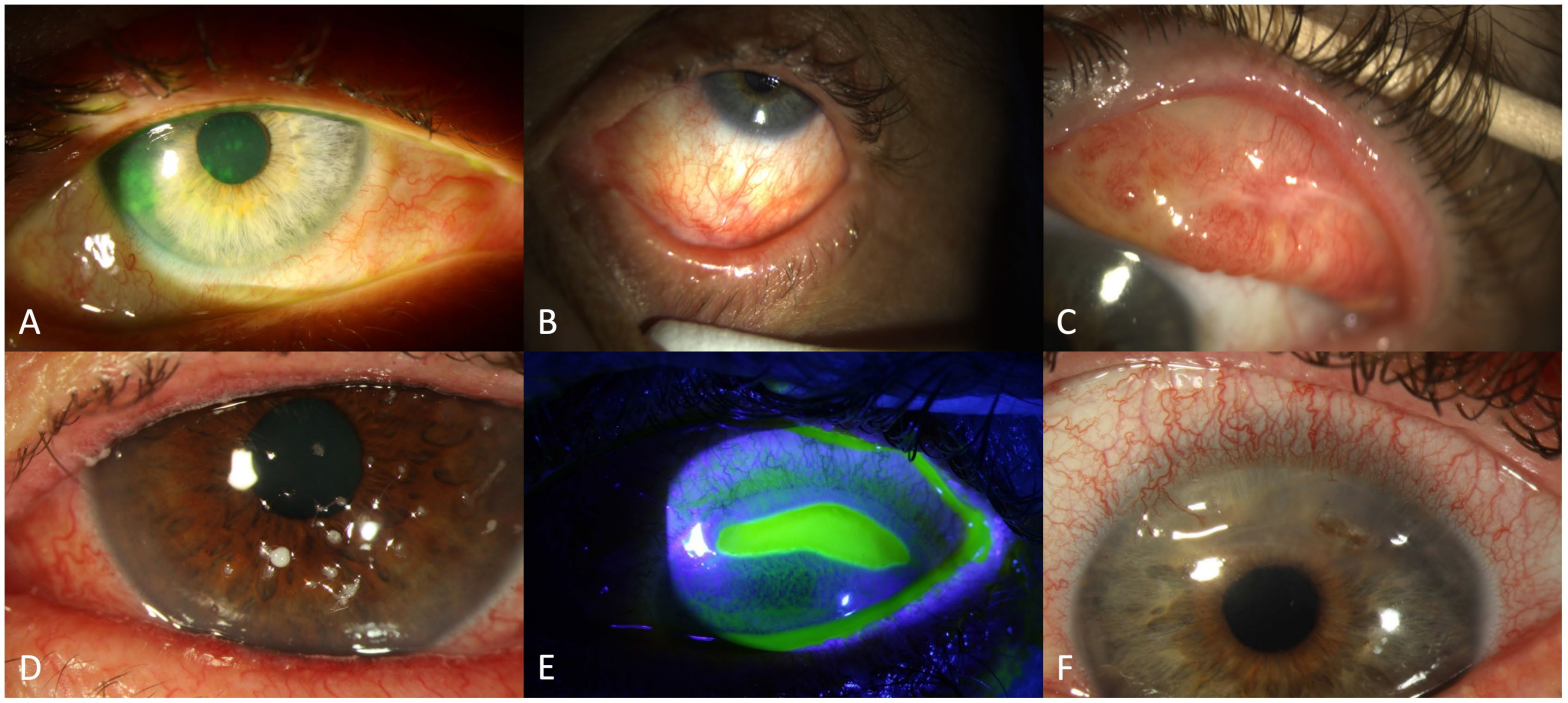
Findings in oGVHD: conjunctival hyperaemia and corneal staining **(A)**, conjunctival scarring/fibrosis **(B)**, conjunctival hyperaemia and symblepharon **(C)**, filamentary keratitis **(D)**, sterile corneal ulceration **(E)** and corneal melting with perforation **(F)**.

Conjunctival involvement mostly manifests as hyperemia (Figures 2A,B) and chemosis. Qualitative and quantitative alterations of the tear film are common, probably with serosanguineous exudation (78). In severe course, pseudo-membrane formation may be observed. Conjunctival fibrosis and subsequent scarring (Figures 2B,C) may not only result in loss of goblet cells, but also to entropion, distichiasis and trichiasis. Therefore, thorough subtarsal inspection is mandatory to determine the pathology also under the upper lid. Indeed, subtarsal fibrosis may correlate with worsening of corneal epitheliopathy. Inflammation and staining of the superior tarsal and bulbar conjunctiva with alteration of the superior limbal epithelium may be present (superior limbal keratoconjunctivitis; SLK-like appearance). The ICCGVHD grading system for conjunctival involvement in oGVHD is shown in Table 3 (15, 67).

**Table 3:** Grading of conjunctival disease in ocular graft versus host disease according to the international chronic oGVHD consensus group (15, 67).

Acute (79)	Conjunctival hyperemia (Stage I), hyperemia with chemosis and/or serosanguineous exudates (Stage II), pseudomembranous conjunctivitis (Stage III), pseudomembranous conjunctivitis with corneal epithelial sloughing (Stage IV). Comment to pseudomembranes: Clinically, these are more probable membranes, as basement membrane is disrupted.
Chronic (66)	Grade 1: conjunctival hyperemia occurring on the bulbar or palpebral conjunctiva in at least one eyelid. Grade 2: palpebral conjunctival fibrovascular changes along the superior border of the upper eyelid, or the lower border of the tarsal plate of the lower eyelid, with or without conjunctival epithelial sloughing, involving <25% of the total surface area in at least one eyelid. Grade 3: palpebral conjunctival fibrovascular changes occurring along the superior border of the upper eyelid, or the lower border of the tarsal plate of the lower eyelid, involving 25–75% of the total surface area in at least one eyelid. Grade 4: >75% of the total surface area with or without cicatricial entropion in at least one eyelid

Morphological abnormalities of the cornea involve punctate keratopathy (Figure 2A), erosions, or filamentary keratitis (Figure 2D) in the more severe cases. Further, limbal stem cell deficiency, Bowman abnormalities, stromal thinning, ulceration (Figure 2E), scarring, calcification and neovascularization may appear. Corneal perforation (Figure 2F) may be secondary to epithelial barrier dysfunction and microorganisms (herpes simplex virus or bacteria), or as sterile “melt” probably in the setting of immunosuppression (80). According to previous reports, corneal ulceration or perforation is found in about 5% of cases (69).

Further, signs of episcleritis or scleritis, secondary cataract (10%, mostly from steroids), or glaucoma (also including steroid-induced ocular hypertension) may appear (81). Within a cohort of 635 patients undergoing HCT, 7.6% had secondary posterior eye segment complications, e.g., retinal hemorrhage, cytomegalovirus retinitis, or uveitis (40, 82).

### 6.2. Diagnostic techniques

A thorough ophthalmological examination is essential in patients with (suspected) oGVHD (83). For assessing the course of disease and response to treatment, a standardized documentation of ocular findings should be performed (Table 4). Assessing ocular findings at baseline before HCT and during follow-up visits allow to early detect worsening of the ocular surface (52, 68, 71, 84). A minimal set of data as visual acuity, slit lamp findings and intraocular pressure should be collected at each visit. Further investigations should be performed as appropriate.

**Table 4:** Consensus Conference Proposal for diagnostic measures for assessment of ocular GVHD (75).

Baseline examination after conditioning treatment and before HCT	Visual acuity test, slit-lamp examination including subtarsal inspection and fluorescein staining, Schirmer test, and fundoscopy
Baseline ophthalmological assessment at day 100–200	Visual acuity test, slit-lamp examination including subtarsal inspection and fluorescein staining, and Schirmer test
Ophthalmological assessment if any other manifestation of GVHD or ocular symptoms	Visual acuity test, slit-lamp examination including subtarsal inspection, vital dyes, Schirmer test, additional tests if indicated (e.g., tear film breakup time), tonometry, and fundoscopy
Routine ophthalmological assessment for 5 years after HCT	Including Schirmer test and glaucoma and cataract assessment
Conjunctival biopsy	Indicated in individual or uncertain cases (e.g., ocular signs or symptoms with no other documented GVHD) or in clinical studies

Diagnostic measures should be adapted to the patient’s overall condition and age; for example, in general, the Schirmer test should not be performed in children.

The Schirmer test I (without topical anesthesia) and II (with prior topical anesthesia) allows to assess the tear production during a defined time of 5 min. A folded filter paper strip is placed in the temporal third of the lower lid margin and the length of the wetting is measured (52). The Schirmer test without anesthesia is also included in the oGVHD (ICCGVHD) consensus group diagnostic criteria (67). While the Schirmer test is useful for diagnosing disease, it was removed from scoring recommendations, as values were not useful for follow-up due to poor correlation with symptom change (64). Due to its low reproducibility it has been removed in the revision of the 2005 NIH criteria and not been included in the 2014 NIH severity scoring, nor in the 2016 Japanese and Asian diagnostic criteria for dry eye disease (23, 63, 85).

Esthesiometry allows to assess the corneal sensitivity, which may be decreased due to pre-conditioning irradiation and neurotrophic keratopathy in patients with oGVHD (86–89).

**Figure 3:**
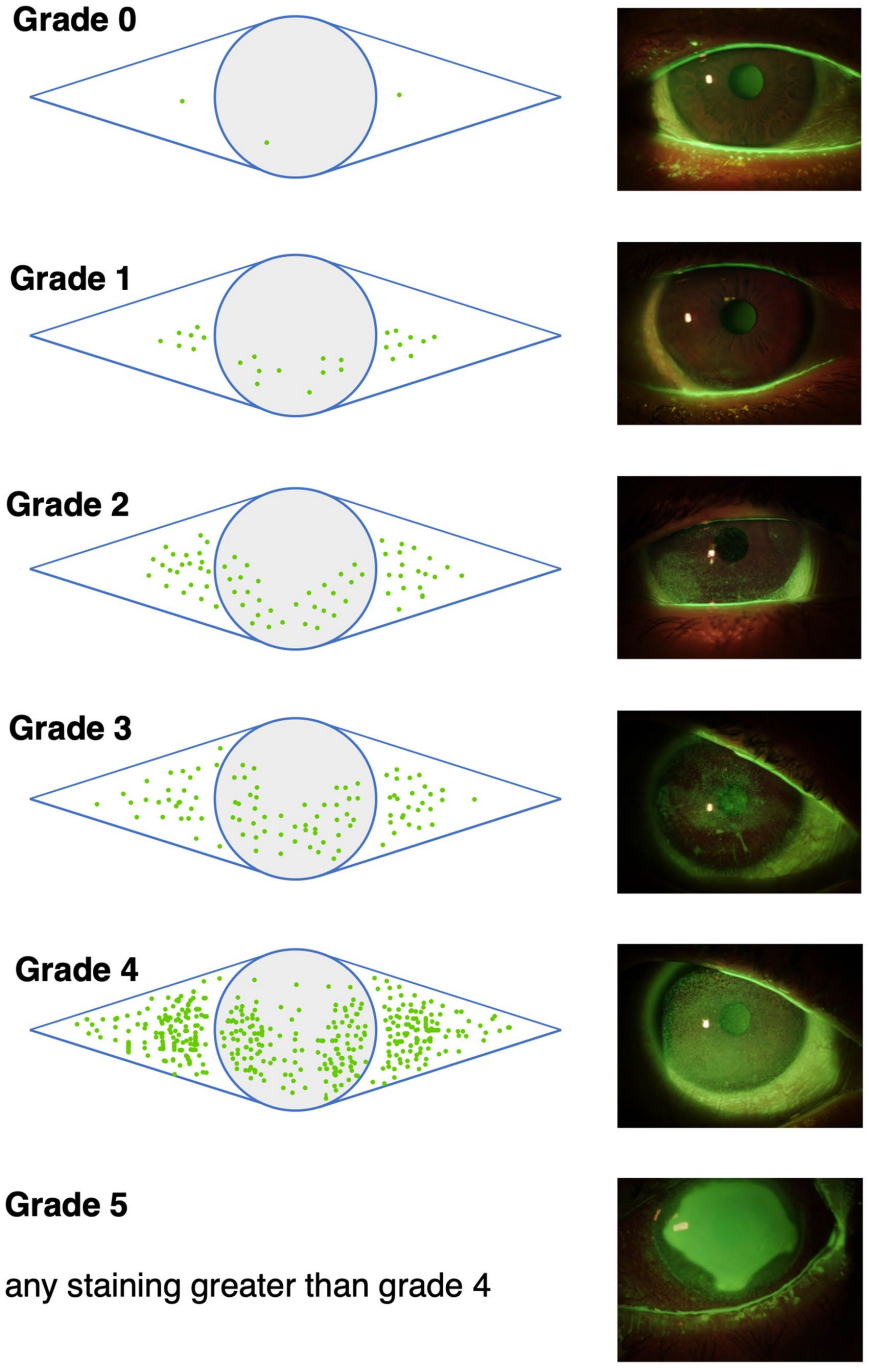
The Oxford grading scheme differentiates 5 grades of corneal and conjunctival fluoresceine staining. Image adapted from Bron et al. (90).

**Figure 4:**
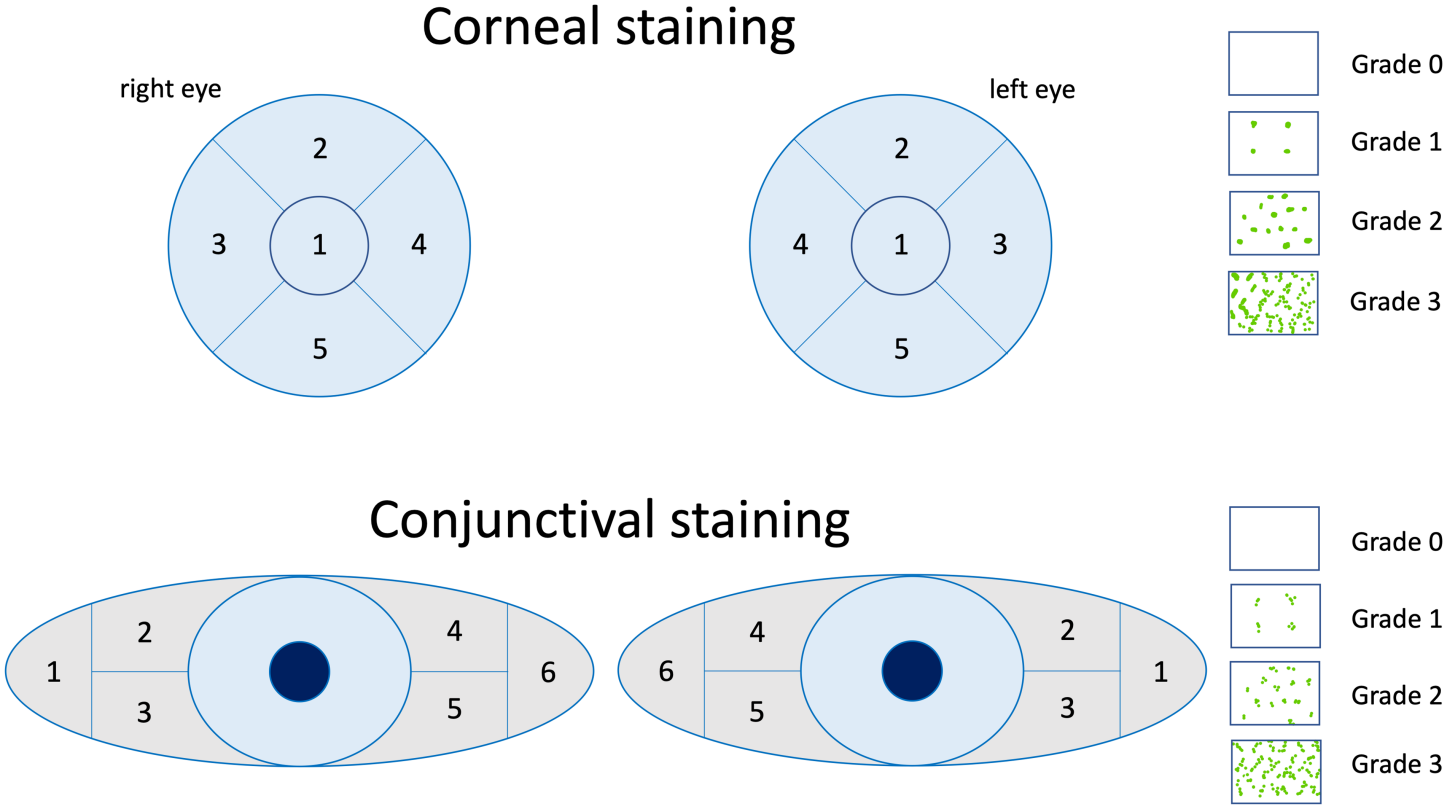
The NEI grading for corneal and conjunctival staining of the ocular surface is a standardized grading system that is summed up by the grading of 0 to 3 of each sector. Image adapted from Lemp et al. (91).

Impairment of conjunctival and/or corneal epithelial integrity can be depicted with vital dye staining. Fluorescein is commonly used to evaluate the corneal staining according to the Oxford grading scheme (Figure 3) and/or the NEI grading for corneal and conjunctival staining (Figure 4) (52, 74, 92). Fluorescein dye is disclosing any disruption in superficial cell tight junctions, or defective glycocalyx of damaged epithelial cells (52). Additional dyes as Bengal rosa or lyssamine green can additionally be used in selected patients (90).

After fluorescein installation, the tear film break-up-time (TBUT) can be evaluated at the slit lamp (52, 70). A decreased TBUT indicates qualitative tear film impairment primarily due to Meibomian gland dysfunction (93).

Tear film osmolarity measurements reveal increased values in oGVHD (74, 94–96) and may be used as an additional factor in therapeutic decisions (19). The tear film osmolarity is also used in the ICCGVHD criteria (23, 95).

Meibomian gland imaging enables the assessment of Meibomian glands, which are often impaired in patients with oGVHD (52, 97–99).

In patients with keratitis, viral and/or microbial tests from corneal smears should be considered to identify viral (mainly by herpes simplex or varicella zoster virus), bacterial or fungal keratitis. The risk for infectious keratitis may be increased in patients under corticosteroid treatment.

*In vivo* confocal microscopy can be used as a diagnostic tool in patients with oGVHD to image epithelial cell density, epithelial dendritic cells and other inflammatory cells, subtarsal fibrosis and conjunctival changes (23, 52, 59, 99–103).

The use of anterior segment photography may be considered to document ocular findings (e.g., staining of ocular surface, conjunctival scarring/fibrosis, blepharitis). It may especially be useful for follow-up comparison of clinical course (75, 104).

Conjunctival impression cytology enables identification of epithelial cell necrosis, keratinization, goblet cells loss and also HLA-DR expression (83, 105, 106). As an alternative, Brush cytology is also a minimally invasive procedure to harvest ocular surface epithelium and inflammatory cells and to monitor pathological progress (88, 107), but interpretation might be difficult due to mechanical alteration of the harvested cells.

Tear film biomarkers (cytokines) can either directly be measured with specific antigen tests (e.g., MMP-9) (108) or (currently mainly for research purpose and not in clinical routine) by performing proteomics from tear fluid or tear-film soaked Schirmer stripes (52, 109). In eyes with oGVHD a variety of cytokines are differently expressed. Especially nucleic acid binding and cytoskeletal proteins are upregulated, while the most extensively downregulated proteins belong to an array of classes including transfer and receptor proteins, enzyme modulators, and hydrolases (109).

Tear flow cytometry is a novel approach, currently used mainly for research purpose, that allows differentiation of cells non-invasively from tear samples (51).

Histopathology may confirm the diagnosis of oGVHD. However lacrimal gland biopsies should not be performed routinely due to the increased risk of further impairment of its function. Previous investigations found mononuclear infiltration, loss of acinar lobules and fibrosis of the lacrimal gland in oGVHD (11, 110, 111). Also, conjunctival biopsies are not performed routinely but may be considered in selected patients, e.g., to rule out malignancy. In conjunctival specimen of oGVHD, lymphocyte exocytosis, vacuolization of the basal epithelium, and epithelial cell necrosis, similar to changes that are observed in other organs, have been found (11, 110, 111). Furthermore, T cells—probably driving alloreactivity in GVHD—have been found in conjunctival biopsies (112).

### 6.3. Questionnaires

Ocular surface inflammation and dryness may have a relevant impact on the quality of life and activities of daily living in patients with oGVHD (22). Different validated questionnaires are used to quantify symptoms, to assess the burden of disease and to track response to treatment (11, 22). The Ocular Surface Disease Index (OSDI), consisting of 12 patient-related questions of dry eye, and the Symptom Assessment in Dry Eye (SANDE) are commonly used questionnaires to assess symptoms in these patients (22, 72, 113–115). Alternatively, or additionally, the glaucoma symptom scale (GSS) may be used (116). On the other hand, the National Eye Institute Visual Function Questionnaire (NEI-VFQ-25) allows to assess vision related quality of life (22). Saboo et al. evaluated patients with oGVHD using the NEI-VFQ-25, OSDI and SANDE questionnaires and found a relevant impact of this disease on quality of life, that is comparable to other eye diseases as for example herpetic uveitis (22).

## 7. Treatment of ocular GVHD/management of complications

The primary aim of treating oGVHD is to maintain vision and quality of life by improving lubrification of the ocular surface (tear film quantity and quality), reducing ocular surface inflammation and preserving corneal epithelium integrity (5). The evidence for different treatments has recently been reviewed by Inamoto et al. (52).

### 7.1. Lubrication

An intensive lubrication for dry and inflamed ocular surface is essential in oGVHD (5, 83, 117). A variety of artificial tears, viscous eye drops, and viscous ointments are available and only limited data on specific preferences for oGVHD is available. In any case, preservative-free formulations should be preferred to avoid the negative impact of preservatives on the epithelium, especially if applied at high frequencies (118). Hyaluronic acid eye drops allow stabilization of the tear film and improvement of epithelial wound healing, ocular symptoms, and visual acuity (53, 117). Increasing the lubrification may also reduce the concentrations of proinflammatory cytokines on the ocular surface (5, 119). Mucolytic eye drops, i.e., topical N-acetylcysteine 5%–10%, should be considered in filamentary keratitis, which is often observed in eyes with a very dry ocular surface (5, 120).

### 7.2. Topical anti-inflammatory treatment

Reducing ocular surface inflammation is a key concept in the management of oGVHD. Topical corticosteroids are effective in treating dry eye in these patients (52, 66, 75). However, due to their probable adverse effects and risks, their application over a longer time periods, or at high dosages and/or with highly potent formulations should be avoided, or regular ophthalmological checks (intervals depending on corticosteroid dosage and duration, eye pressure and lens status) be instituted. Potential risks include cataract formation, infections, ocular hypertension/glaucoma, impaired epithelialization and impaired corneal wound healing (5, 11, 66). Nevertheless, they are used commonly in oGVHD patients (5, 52, 121, 122). However, topical corticosteroids are not able to sufficiently control oGVHD in about half of the patients (7). Low-dose/−less potent topical corticosteroids or their analogs seem to be less effective in patients with oGVHD compared to dry eye patients without oGVHD (11, 123). As an anti-inflammatory treatment option, cyclosporine (CsA) eye drops are used in patients with treatment refractory dry eye disease. CsA acts as a calcineurin inhibitor and suppresses T-cell activation (11, 124), and its efficacy has also been proven in patients with oGVHD (11, 125). Hereby, it reduces ocular surface inflammation, increases conjunctival goblet cell density and tear production and improves symptoms of dry eye (5, 75, 125–129). If treatment is initiated before HCT, it probably reduces the risk for oGVHD manifestation (130). However, a reduced tolerance (burning sensation) of topical CsA may limit its use in some patients. Furthermore, tacrolimus eye drops or ointment have been studied in patients with oGVHD, probably allowing corticosteroid sparing (11, 131–133). Tacrolimus ointment may also be applied to the eyelids as an off-label treatment. Although topical non-steroidal anti-inflammatory drugs (NSAIDs) are also used in oGVHD, there is no evidence for their efficacy.

### 7.3. Autologous serum eye drops

Based on several uncontrolled trials in oGVHD and in analogy to other forms of dry eye disease, autologous serum eye drops are also used in patients with oGVHD, especially in severe cases (86, 117, 134). Although the exact mechanism of action is not known, the high concentration of several growth factors combined with anti-inflammatory effects are suggested to improve healing of epithelial defects (129, 135, 136). Systemically applied cyclosporin A or mycophenolic acid might also be detectable in serum eye drops (137) and could contribute to the observed beneficial effect. Patients impaired condition to donate blood (poor venous access, severe anemia, active infection, low body weight, cardiovascular comorbidities) as well as regulatory restrictions are potential obstacles that prevent access to this therapy. Other options that have been reported are allogeneic serum eye drops (136), cord blood sera (117, 138, 139) and platelet lysate (116, 140). None of these options have become more widely available yet due to a couple of logistics and regulatory reasons.

### 7.4. Control of evaporation

Improving the lipid layer of the tear film with viscous eye drops and ointments, improving the Meibomian gland outflow with eyelid massage and eventually lipid sprays reduce evaporation of the tear film. The evidence for eyelid massage in oGVHD is low and the mechanical friction might even be counterproductive in oGVHD with affection of the corneal epithelium. Occlusive eye wear (52, 141) and an improvement of environmental factors as air humidity may also be helpful (117, 142).

### 7.5. Increase of tear and mucin production

Systemic treatment with oral muscarinic agonists as pilocarpine or cevimeline may increase tear production (117, 143, 144). As adjuvant treatment approaches, secretagogue eye drops as diquafosol and rebamipide may be used in patients with oGVHD (52, 101, 145). They stimulate secretion of aqueous and mucin and improve wound healing of the corneal surface (5, 101, 146).

### 7.6. Reduction of tear drainage

**Figure 5:**
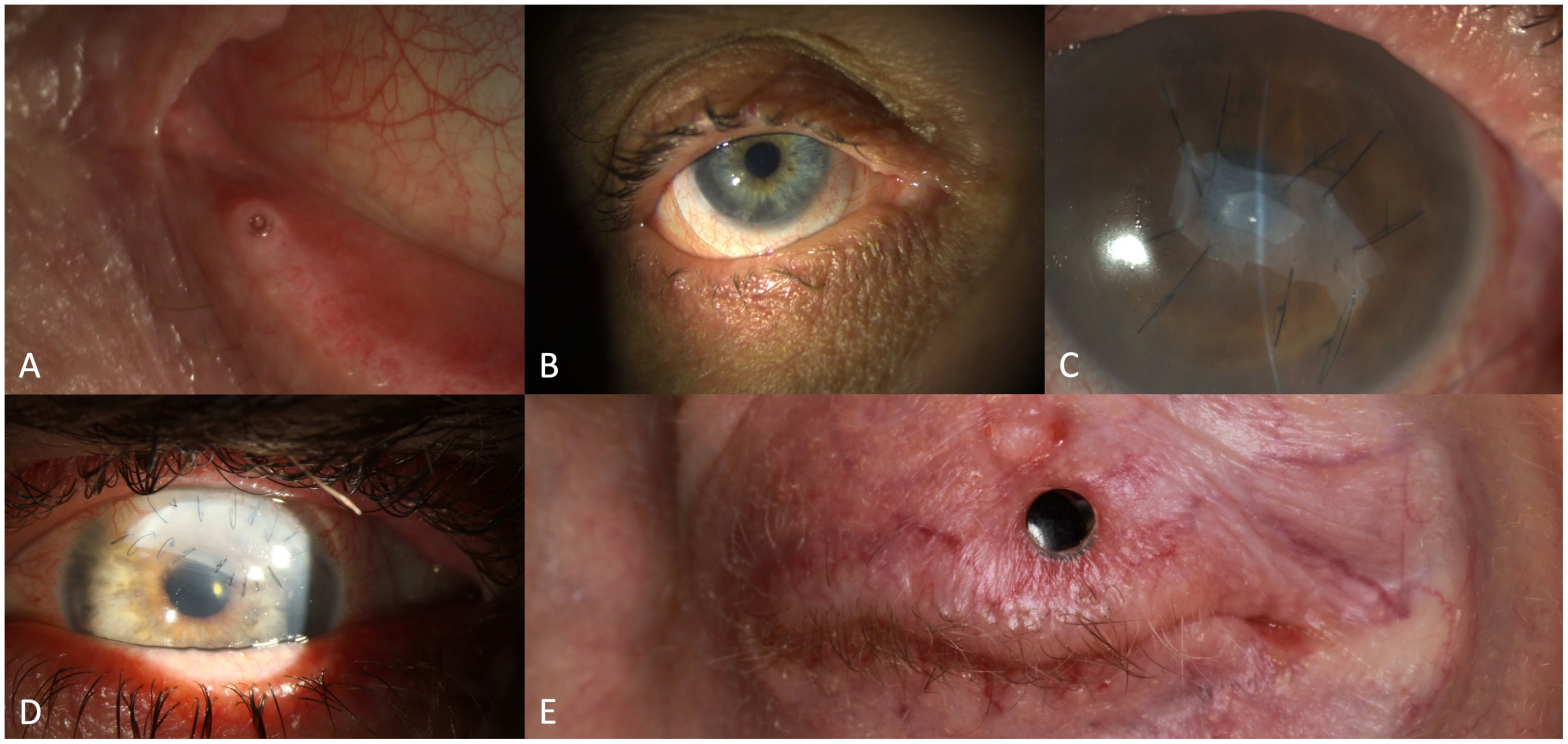
Therapeutic interventions in eyes with oGVHD: silicone punctal plug **(A)**, scleral lens **(B)**, amniotic membrane transplantation **(C)**, lamellar keratoplasty with loosening of the sutures **(D)**, transpalpebral osteo-odonto-keratoprosthesis **(E)**.

Reduction of the lacrimal drainage is a further approach to improve the tear film (11). Here, collagen or silicone punctal plugs (Figure 5A) may be inserted into the lacrimal ducts, or permanent punctal occlusion by thermal cauterization may be considered (147, 148). It has been speculated that reducing the tear drainage might result in a pooling of pro-inflammatory cytokines and increase damage of the ocular surface and patient discomfort (149). Positive effects of punctal occlusion predominate in the clinical situation (86, 147).

### 7.7. Scleral lenses

The use of scleral lenses (Figure 5B) in patients with severe oGVHD has been shown to reduce ocular symptoms and especially ocular pain (83, 150) and improve visual acuity due to their uniform surface (11, 83, 107, 119, 150–155). These gas-permeable lenses cover most of the ocular surface, vault the cornea and limbus providing a fluid reservoir between the cornea and the lens (83). Furthermore, they protect the ocular surface from mechanical “scratching” from blinking (83). In a study by Schornack et al., most patients were still on scleral lenses after a 32-month observation period, indicating a relevant patient satisfaction (152). High costs, inadequate fitting, discomfort with blinking may be potential drawbacks (119). As an alternative to scleral lenses also soft contact lenses have been investigated in oGVHD (156), but may potentially bear a higher risk of infection (83).

### 7.8. Prevention of infectious disease

Especially in eyes with severe oGVHD, epithelial defects or even corneal melting may occur, due to the very dry and inflamed ocular surface. In this situation, infectious prophylaxis with topical antibiotics should be taken into consideration (19). In patients with extended wear of contact lenses (especially soft contact lenses and topical corticosteroid treatment) topical antibiotic prophylaxis should be considered (157). Furthermore, topical antibiotic ointments or eye drops but also systemic tetracyclines (e.g., doxycycline or minocycline) may be considered in patients with blepharitis as a sign of bacterial superinfection of the eyelids (11, 52, 75, 158).

### 7.9. Systemic treatment

Systemic treatment of oGVHD is absolutely indicated if severe oGVHD cannot be controlled with topical treatment alone.

High dose corticosteroids (methylprednisolone 1 mg/kg) remain the mainstay of initial systemic treatment of chronic GVHD, either given alone or in combination with calcineurin inhibitors, especially in high-risk disease (159). Second line treatment is indicated in case of steroid-refractory chronic GHVD with an increasing number of treatment options (160). Up to now, there is no standard yet (161). Levels of evidence for efficacy and treatment costs vary considerably and numbers of patients reported for eye response are usually low (162). Extracorporeal photopheresis (ECP) has been reported to resolve or improve eye manifestation in 30% compared to 7% with standard therapy alone by Flowers et al. (163). Other studies could confirm these results in similar or higher magnitude. Recently, ruxolitinib (Janus kinase 1/2 inhibitor; FDA and EMA) and belumosudil (inhibitor of Rho-associated coiled-coil-containing protein kinase 2; FDA) have been approved for treatment of steroid-refractory chronic GVHD. Both have been shown to be effective in a proportion of patients with oGVHD. In the randomized open-label REACH3 trial overall response was 26% with ruxolitinib versus 10.8% with best available treatment (164). Belumosudil was studied in the phase 2 ROCKstar trial mainly in patients with advanced, steroid-refractory chronic GVHD with a remarkable overall response rate of 42% (14% complete responses, 28% partial remissions) (165). In contrast, there are no conclusive data with the third FDA-approved agent ibrutinib (inhibitor of Bruton’s tyrosine kinase) in oGVHD (166). Other agents that are frequently used are sirolimus (mTOR inhibitor), bortezomib (proteosome inhibitor), imatinib (tyrosine kinase inhibitor) and low-dose methotrexate (162). However, there are no randomized controlled trials that evaluated the effect of systemic treatment specifically on oGHVD, or that investigated superiority of one agent to another.

Several new systemic therapeutic principles are tested in preclinical studies including bromodomain inhibitors (167) and SYK inhibition by entospletinib (168).

### 7.10. Antifibrotic treatment

Currently, no specific treatment strategy is available for fibrosis. Given the pathophysiology of chronic oGVHD, anti-inflammatory and anti-fibrotic treatment regiments might be beneficial. Topically, corticosteroids may have some local antifibrotic effect, but clinical relevance is unknown, and risks do not justify prolonged application. TGF-b signaling inhibition (tranilast) may be useful (169, 170). In contrast to topically applied agents, systemic DMARDs therapy is commonly recommended for severe oGVHD not properly responding to topical agents, as untoward side effects may occur. Agents such as corticosteroids and steroid sparing agents may be applied, including ciclosporin, tacrolimus, sirolimus, mycophenolate mofetil, and particularly B cell blockade with rituximab. Case reports document the value of amniotic membrane transplantation (AMT) for preventing excessive fibrosis (171).

## 8. Surgical management of complications

No data exist on how often surgical treatment for complications of chronic oGVHD is necessary. This section gives an overview of different surgical interventions for the most common complications of oGVHD.

### 8.1. Cauterization of lacrimal punctum

Punctal occlusion with punctal plugs has been shown to be safe to treat severe dry eye in oGVHD (147) and is often used. In rare cases plugs are not supported or extruded repeatedly. In such situations permanent surgical occlusion is possible. Yaguchi et al. described their method of punctal cauterization with a high-temperature sterile disposable cautery device in 23 puncta from 10 oGVHD patients (148). They achieved a 100% anatomical success without recanalization after 1 year and reported no surgical complications. Several other methods for surgical punctal occlusion in other etiologies of dry eye disease have been described, including thermal cautery, diathermy, laser coagulation and punctal suturing (172–177).

### 8.2. Tarsorrhaphy and botulinum toxin

Inflammation and tear deficiency in oGVHD can lead to severe corneal ulcerations (87, 178). In such situations, temporal or complete temporary tarsorrhaphies or botulinum toxin A induced protective ptosis (179) are good options to protect the cornea and gain time when systemic immunomodulatory treatment is initiated or escalated and not fully effective yet. Yeh et al. described a patient with oGVHD in whom even tarsorrhaphy and amniotic membrane transplantation (AMT) were not enough, and eventually the eye had to be eviscerated (180).

### 8.3. Amniotic membrane transplantation, cyanoacrylate glue or conjunctival (Gundersen) flap

Amniotic membrane transplantation (Figure 5C) is a surgical procedure that may help to prevent or stop corneal melting by reconstructing the ocular surface and supporting the epithelialization of the cornea (181–184). Epithelial recovery and suppression of inflammation may be achieved due to the contained cytokines and growth factors, additionally the amnion membrane acts as a mechanical barrier for frictional forces (184–187). Indeed, AMT has also successfully been used in progressive corneal ulcers in oGVHD patients (171, 188–190). However, only limited data are available about its success rate up to now. In deep corneal ulcers or descemetocele with pending perforation, cyanoacrylate glue may be an option to avoid or delay more invasive corneal surgery (80, 189). Conjunctival (Gundersen) flap may be another option to cover a corneal ulcer or a fresh corneal transplant. Xu et al. described four oGVHD patients in whom they combined tectonic penetrating keratoplasty with conjunctival flaps (191). Furthermore, Pellegrini et al. reported on one patient receiving a Gundersen flap for impending perforation in their case series of 283 patients with HCT (192).

### 8.4. Keratoplasty and keratoprosthesis

Despite intensive topical and systemic treatment and tarsorrhaphy and/or AMT, corneal perforations might still occur in severe oGVHD. In such situations, keratoplasties might be required. One option is to perform an urgent tectonic keratoplasty (Figure 5D) with the primary aim of saving the eye and gaining time to escalate the anti-inflammatory treatment. Another possibility is to perform a penetrating keratoplasty with the aim of restoring vision and globe integrity at the same time. Corneal transplant diameters from only few millimeters to large may be used for such keratoplasties depending on the individual need. Sinha et al. determined that the prevalence of corneal perforation in patients with oGVHD was 3.7% (193). Zhang et al. reported 14 corneal perforations in patients with oGVHD during an observation period of 59 years at 4 large centers (80). They all were initially glued and 8 needed penetrating keratoplasty, which had diameters of 2 to 9.5 mm. The best corrected visual acuity outcomes at last visit were 20/100 or better in 5 patients (36%), and hand motion or worse in 7 patients (50%) (80). Xu et al. reviewed 198 oGVHD patients within an observation period of 9 years and identified 9 eyes of 7 patients with corneal perforation necessitating penetrating keratoplasty (trepanation diameters of 2 to 8 mm were used). Only two eyes of two patients achieved a final best corrected visual acuity of 20/100 or better (191). Sometimes even repeat keratoplasty cannot prevent perforations and re-establish functional visual acuity, reason why we had to perform a through-lid Osteo-Odonto-Keratoprosthesis (OOKP; Figure 5E) in one patient (194) and Osteo-Keratoprosthesis (OKP) in another. The outcome was successful in both patients with a best corrected visual acuity of 20/32 or better. Liu et al. mentioned one oGVHD patient in their 10-years review on 36 patients with OOKP (195). Furthermore, Orive Bañuelos et al. also described an oGVHD patient who received a Boston keratoprosthesis Type II after several corneal perforations with repeated keratoplasties. As a further complication, probably related to the keratoprosthesis surgery, two cyclophotocoagulations had to performed. The final visual acuity was 20/20 but the visual field revealed glaucoma related damage (196). OOKP and OKP are high risk procedures that are not commonly performed but might sometimes be the last resort to restore vision in selected patients.

### 8.5. Cicatricial entropion repair and fornix reconstruction

Chronic conjunctival inflammation and subepithelial fibrosis, are often found in oGVHD and can eventually lead to progressive conjunctival scarring with entropion and trichiasis. In combination with keratoconjunctivitis sicca these complications can be devastating for the ocular surface, reason why cicatricial entropion and trichiasis have to be treated without delay (197). Komai et al. described the cultivated oral mucosal epithelial transplantation (COMET) as a method to treat fornix shortening/symblepharon in different chronic cicatrizing conjunctival diseases (198). One of their patients suffered from oGVHD and was successfully treated with this surgical method (198). Dulz et al. described a 7-year-old boy who developed a massive bilateral cicatricial entropion with trichiasis 5 years after HCT. They performed bilateral lamellar splitting *via* an eyelid crease and gray line incision. Cryocoagulation of persistent trichiatic lashes was additionally performed (199). Kheirkhah et al. utilized a combined approach with mucous membrane transplantation from the lower lip covering it with AMT for their series of symblepharon, among which was also a successfully treated oGVHD patient (200).

### 8.6. Cataract surgery

Cataracts frequently develop in patients after HCT. This is probably a side effect of the treatments with corticosteroids or total body irradiation, and not due to GVHD directly. The long-term use of topical corticosteroids, particularly when given at higher dosages increases the risk for cataract formation. In patients with oGVHD inactivity of the ocular surface inflammation and optimal stabilization of the dry eye disease is required before surgery (201), and a good peri-operative management is critical. Bae et al. described 77 cataract surgeries in 42 patients suffering from oGVHD. Out of these patients, 19 postoperatively developed punctate keratopathy, that was being treated with artificial tears or autologous serum drops; another 7 eyes developed corneal epithelial defects, requiring non-steroidal anti-inflammatory eye drops, and another 3 eyes had cystoid macular edema (202). These findings are supported by others, additionally reporting on corneal melts and perforation after surgery (203–207). Taken together, oGVHD patients require close post-operative monitoring and prolonged anti-inflammatory treatment.

## 9. Novel approaches and outlook

As described previously, the clinical manifestations of oGVHD are the result of various structural and functional changes in lacrimal and Meibomian glands, eye lids, quantitative and qualitative alterations of the tear film and damage of the ocular surface. It is likely that the contribution of each of this component to active oGVHD differs between individuals. Symptoms might manifest after the damage has already been set. Hence, a standardized ocular assessment and documentation as part of the posttransplant follow up as well as the identification of specific biomarkers might allow a better understanding of the pathophysiology of oGVHD, an earlier diagnosis in the future (47) and potentially also to identify eyes at risk for severe complications. Ophthalmologists should be constant members of multidisciplinary teams providing posttransplant care. More efficient treatments that prevent or treat inflammation and enable regeneration of the dysfunctional ocular surface, lacrimal glands and Meibomian glands are needed. Pre-clinical animal models of GVHD enable developing and investigating new treatments (208). During the last decade, the number of interventional studies in oGVHD has slowly increased. Most of them are single center trials of topical treatments involving limited patient numbers. Randomized controlled trials of topical and systemic treatment options in patients with oGVHD are urgently needed and could expand our current knowledge considerably.

Anti-inflammatory drugs as tocilizumab and sarilumab, that impact the IL-6 pathway, are promising as they have been shown to be beneficial in animal models of oGVHD (117, 209, 210). Furthermore, Janus kinase (JAK) inhibitors either alone or in combination with tyrosine kinase (SYK) inhibition are a further interesting option as an early intervention that had a favorable effect in a pilot study (211). Belumosudil is another promising new approach even in heavily pretreated chronic GVHD (165). It will be important to study the therapeutic potential of this drug on oGVHD in earlier lines of treatment because of its anti-inflammatory and antifibrotic action.

Innovative options coming from basic research and/or animal studies, like ATR type I antagonist, VAP-1 inhibitor, phenyl butyric acid, tranilast, heavy chain-hyaluronan/pentraxin 3 (HC-HA/PTX3), ABT-263 and vitamin A-coupled liposomes containing HSP4 siRNA reversed the changes seen in oGVHD (117). In a pilot trial pooled human immunoglobulin eye drops were promising for treating oGVHD (212). A variety of further ongoing trials in oGVHD investigate the potential of other therapeutic approaches, e.g., topical fibrinogen-depleted human platelet lysate, brimonidine nanoemulsion, rhDNase eye drops as well as different types of contact lenses (23).

## 10. Conclusion

A better understanding of the pathophysiology of oGVHD, definition of standardized diagnostic criteria, introduction of grading systems, increasing experience with different topical and systemic treatments, but also with tools as, e.g., punctal plugs or scleral lenses, has improved the management of this disease. Nevertheless, oGVHD still has a relevant impact on the quality of life of HCT survivors. Severe and potentially blinding complications as corneal perforations cannot always be prevented. There is a high need for randomized controlled trials comparing the efficacy of different treatment regimens and supporting measures.

## Author contributions

CT, AH, and DG contributed to conceptualization. CT, AH, and JH performed the literature research. CT, AH, JH, and DG wrote individual chapters of the manuscript. DG, CT, and EM provided slit lamp photographs. CT produced the figures. All authors contributed to the article and approved the submitted version.

## Funding

Open access funding was provided by University of Bern.

## Conflict of interest

The authors declare that the research was conducted in the absence of any commercial or financial relationships that could be construed as a potential conflict of interest.

## Publisher’s note

All claims expressed in this article are solely those of the authors and do not necessarily represent those of their affiliated organizations, or those of the publisher, the editors and the reviewers. Any product that may be evaluated in this article, or claim that may be made by its manufacturer, is not guaranteed or endorsed by the publisher.
